# Tourette’s syndrome: Alternative approaches to a clinical case refractory to conventional therapy

**DOI:** 10.1192/j.eurpsy.2021.1309

**Published:** 2021-08-13

**Authors:** M.I. Fonseca Marinho Vaz Soares, S. Freitas Ramos, D. Cruz E Sousa, B. Jesus, S. Castro

**Affiliations:** Psychiatry And Mental Health, Hospital, Guarda, Portugal

**Keywords:** Deep brain stimulation, antiglutamatergic, Tourette syndrome, Tics

## Abstract

**Introduction:**

Tourette’s syndrome is a neuropsychiatric disorder marked by motor and phonic tics frequently associated with psychiatric comorbidities, beginning in childhood. While most cases improve or resolve with age, some are refractory.

**Objectives:**

To review new strategies for the management of Tourette’s Syndrome, following an outpatient clinical vignette.

**Methods:**

We performed a review based on the PubMed® database.

**Results:**

A 50-years-old female patient with a long-term outpatient psychiatric follow-up presented with motor tics appearing in adolescence, including winking and facial grimacing, as well as episodes of coprolalia. Over the years, she developed an anxiety disorder and social isolation. In addition to psychological therapy, pharmacological therapy had already been approached with the use alpha-adrenergic agonists and several antipsychotics, such as risperidone and aripiprazole, with the patient showing only partial response to pimozide. In Tourette’s syndrome, the therapy must be adequate to the patient’s individual needs. Emerging treatments for refractory cases, such as anticonvulsants, cannabinoids or antiglutamatergic drugs have been the target of several studies. Botulinum toxin injections are particularly effective in patients with focal motor tics and complex phonic tics. Non-pharmacological treatment options, such as electroconvulsive therapy and deep brain stimulation may prove effective in some cases.

**Conclusions:**

A significant proportion of patients fail to respond to conventional strategies. Thus, new pharmacological and non-pharmacological therapies are on the horizon and may represent an important step in treatment algorithms for refractory cases in the future.

